# Osteolectin^+^ stromal cells: Mechanical stimulation improves bone regeneration and supports bacterial clearance after fracture

**DOI:** 10.1038/s41392-021-00680-7

**Published:** 2021-07-07

**Authors:** Sabrina Ehnert, Tina Histing, Andreas K. Nüssler

**Affiliations:** Department of Trauma and Reconstructive Surgery Eberhard Karls University Tübingen, Siegfried Weller Research Institute, BG Trauma Center Tübingen, Tübingen, Germany

**Keywords:** Stem-cell research, Lymphocytes, Stem-cell niche

In a study recently published in *Nature*, Shen and colleagues characterized a peri-arteriolar niche in the bone marrow with highly proliferative, short-lived, leptin receptor- and osteolectin (Oln) positive stromal cells, which improved bone regeneration and supported bacterial clearance after fracture. By proving that maintenance of these cells critically required mechanical stimulation, which in turn was affected by age,^1^ the authors identified possible therapeutic targets for bone healing and regeneration.

Bone marrow stromal cells (BMSCs), also known as skeletal or mesenchymal stem cells, not only function as adipogenic, chondrogenic or osteogenic progenitors, but also provide cytokines critically required for maintenance and fate of hematopoietic stem cells (HSCs). These cytokines include, among others, stem cell factor (SCF), stromal cell-derived factor 1 and pleiotrophin. In other studies, it was shown that BMSCs producing these cytokines express leptin receptor (LEPR). In the here presented study, the authors showed that the differentiation potential of these LEPR^+^ BMSCs, however, was dependent on the expression of Oln, also known as stem cell growth factor (SCGF) or C-type lectin domain family 11 member A (CLEC11A). Oln was previously shown to be expressed in hypertrophic chondrocytes, osteoblasts, and osteocytes. Oln-negative LEPR^+^Oln^−^ BMSCs preferably differentiated into adipocytes, but retained their osteogenic potential. Oln-positive LEPR^+^Oln^+^ BMSCs were primed towards osteogenesis (Fig. 1).^2^

**Fig. 1:**
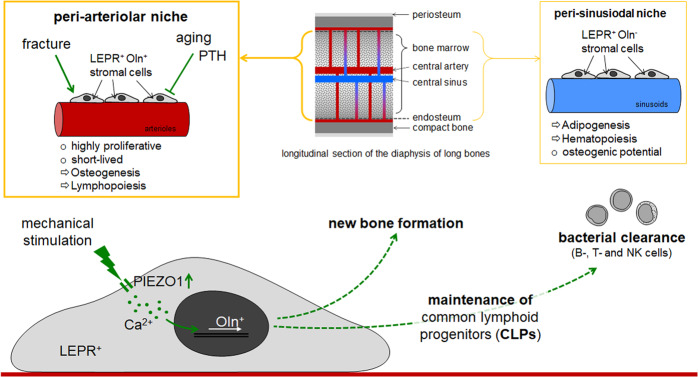
The peri-arteriolar niche is mainly found in the central bone marrow and near the endosteum of diaphyseal bone. In close proximity to the arterioles, the niche harbors highly proliferative, short-lived, stem cell factor (SCF) expressing, leptin receptor (LEPR), and osteolectin (Oln)-positive stromal cells. These cells decrease in number with age and after PTH treatment, but increase in number after fracture and due to mechanical stimulation. A critical regulator for the mechanosensing is the ion channel PIEZO1. The LEPR^+^Oln^+^ stromal cells regulate not only new bone formation, but also maintenance and commitment of common lymphoid progentiors and thus, contribute to bacterial clearance after infections

Using different genetically modified mouse models, the authors elegantly compared LEPR^+^Oln^+^ and LEPR^+^Oln^−^ BMSCs. LEPR^+^Oln^−^ BMSCs localized in the periphery of sinusoidal vessels. In contrast, LEPR^+^Oln^+^ BMSCs were found in the periphery of arterioles, mainly in the diaphysis, the central bone marrow and near the endosteum, which defined the niche for these osteoprogenitor cells. Comparing the two cell types, LEPR^+^Oln^+^ BMSCs showed a significantly higher proliferation rate than LEPR^+^Oln^−^ BMSCs. The rapid cell division of LEPR^+^Oln^+^ BMSCs was accompanied by a high recombination efficiency and a short live-span. Despite these differences, LEPR^+^Oln^−^ and LEPR^+^Oln^+^ BMSCs both expressed SCF. Deletion of SCF in LEPR^+^Oln^+^ BMSCs, did not affect the frequencies of HSCs, multipotent-, granulocyte-macrophage-, megakaryocyte-erythrocyte- or common myeloid progenitors, but significantly reduced the frequency of common lymphoid progenitors (CLPs) in the bone marrow (Fig. 1). In line with these findings, deep imaging of the bones found approximately 1/3 of the bone marrow CLPs in close proximity (<5 µm) to the peri-arteriolar LEPR^+^Oln^+^ BMSCs.

The authors followed several approaches to better characterize the biological function of the peri-arteriolar LEPR^+^Oln^+^ BMSCs. With increasing age, the number of LEPR^+^Oln^+^ BMSCs declined, which was accompanied by bone loss and reduced numbers of CLPs. It was assumed that reduction in LEPR^+^Oln^+^ BMSCs contributes to age-dependent bone loss. Interestingly, increasing the bone mass by applying parathyroid hormone (PTH) decreased the number of LEPR^+^Oln^+^ BMSCs and thus CLPs in the bone marrow, which led to the conclusion that increasing bone volume alone was not sufficient to augment these two cell types in the bone marrow. However, the number of LEPR^+^Oln^+^ BMSCs in the peri-arteriolar niche rapidly increased after a bone fracture. Together with the Oln^+^ osteogenic cells within the callus at the fracture site, LEPR^+^Oln^+^ BMSCs formed most of the osteoblasts that contributed to the regeneration of the bone. Thus, LEPR^+^Oln^+^ BMSCs in the peri-arteriolar niche represent an interesting target to be addressed in delayed or non-union fracture healing, often accompanied by complications such as infections. As depletion of LEPR^+^Oln^+^ BMSCs was associated with a decline in CLPs, the authors also addressed this point. Mice with conditional knockdown of osteolectin, failed to increase B-cell and T-cell counts and clear bacteria from the spleen after oral administration of *Listeria monocytogenesis*. After intraperitoneal administration of *Listeria*, this effect was even more pronounced and also involved NK-cells, and cells of the thymus. Thus, cells of the peri-arteriolar niche seemed to be required not only for maintenance of normal numbers of CLPs, but also for augmentation of lymphopoiesis and for clearance of bacteria after acute infection. Therefore, these cells may also prospectively be addressed in the treatment of septic complications.

It is well described that the bone is a mechanosensitive organ. Thus, the authors performed experiments with mechanical stimulation, in order to investigate possible regulatory mechanisms. In aged mice, exercise in a running wheel increased not only bone mass but also the number of LEPR^+^Oln^+^ BMSCs and CLPs in the weight-bearing bones. For the reverse experiments, the authors suspended hindlimbs of young mice to reduce the mechanical loading. After only 2 weeks, bone mineral density, cortical thickness, as well as LEPR^+^Oln^+^ BMSCs and CLPs were significantly reduced in the femurs of the suspended mice as compared to mice with normal activity. Gene-expression profiling and Western blotting identified the mechanosensitive ion channel PIEZO1 as possible regulator in this process. Applied pressure and pharmacological stimulation of PIEZO1 with Yoda1 both induced calcium influx into LEPR^+^Oln^+^ BMSCs. Deletion of PIEZO1 in LEPR^+^Oln^+^ BMSCs abolished this effect. Furthermore, the deletion of PIEZO1 was accompanied by a significant decline in CLPs substantiating the proposed regulatory role of PIEZO1 in the described peri-arteriolar niche. This mechano-dependence could not be shown for the LEPR^+^Oln^−^ BMSCs in the peri-sinusoidal niche (Fig. 1).

In summary, the manuscript provides a detailed characterization of a novel peri-arteriolar niche in the bone, harboring specific (LEPR^+^Oln^+^) mechanosensitive (PIEZO1^+^) BMSCs that control osteogenesis and lymphopoiesis. These specialized cells represent an interesting target to address clinical challenges, such as osteoporosis, delayed or non-union fracture healing, and associated septic complications. For example, transforming growth factor beta (TGF-β), which is well known to control adhesion, proliferation, migration and mechanosensation of osteoprogenitor cells during bone regeneration,^3^ was reported to maintain multipotency and to inhibit cell commitment and expression of Oln.^4^ Oln in turn was reported to suppress expression of fibroblast activation protein, also known as dipeptidyl peptidase-4 or CD26, actively inhibiting osteogenesis.^5^ These examples show that unraveling the underlying molecular mechanisms may provide novel therapeutic targets to support bone regeneration.

## References

[1] Shen B (2021). A mechanosensitive peri-arteriolar niche for osteogenesis and lymphopoiesis. Nature.

[2] Yue R, Shen B, Morrison SJ (2016). Clec11a/osteolectin is an osteogenic growth factor that promotes the maintenance of the adult skeleton. Elife.

[3] Ehnert S (2017). TGF-beta1 impairs mechanosensation of human osteoblasts via HDAC6-mediated shortening and distortion of primary cilia. J. Mol. Med..

[4] Merrick D (2019). Identification of a mesenchymal progenitor cell hierarchy in adipose tissue. Science.

[5] Wei H (2020). Identification of fibroblast activation protein as an osteogenic suppressor and anti-osteoporosis drug target. Cell Rep..

